# Boundary activated hydrogen evolution reaction on monolayer MoS_2_

**DOI:** 10.1038/s41467-019-09269-9

**Published:** 2019-03-22

**Authors:** Jianqi Zhu, Zhi-Chang Wang, Huijia Dai, Qinqin Wang, Rong Yang, Hua Yu, Mengzhou Liao, Jing Zhang, Wei Chen, Zheng Wei, Na Li, Luojun Du, Dongxia Shi, Wenlong Wang, Lixin Zhang, Ying Jiang, Guangyu Zhang

**Affiliations:** 1 CAS Key Laboratory of Nanoscale Physics and Devices, Institute of Physics, Chinese Academy of Sciences, Beijing, 100190 China; 2 School of Physics and Electronic Engineering, Sichuan Normal University, Chengdu, Sichuan 610101 China; 3 International Center for Quantum Materials, School of Physics, Peking University, Beijing, 100871 China; 4 School of Physics, Nankai University, Tianjin, 300071 China; 5 School of Physical Sciences, University of Chinese Academy of Sciences, Beijing, 100190 China; 6 Collaborative Innovation Center of Quantum Matter, Beijing, 100190 China; 7 CAS Center for Excellence in Topological Quantum Computation, University of Chinese Academy of Sciences, Beijing, 100190 PR China; 8 Beijing Key Laboratory for Nanomaterials and Nanodevices, Beijing, 100190 China

## Abstract

Recently, monolayer molybdenum disulphide (MoS_2_) has emerged as a promising and non–precious electrocatalyst for hydrogen evolution reaction. However, its performance is largely limited by the low density and poor reactivity of active sites within its basal plane. Here, we report that domain boundaries in the basal plane of monolayer MoS_2_ can greatly enhance its hydrogen evolution reaction performance by serving as active sites. Two types of effective domain boundaries, the 2H-2H domain boundaries and the 2H-1T phase boundaries, were investigated. Superior hydrogen evolution reaction catalytic activity, long-term stability and universality in both acidic and alkaline conditions were achieved based on a multi-hierarchy design of these two types of domain boundaries. We further demonstrate that such superior catalysts are feasible at a large scale by applying this multi-hierarchy design of domain boundaries to wafer-scale monolayer MoS_2_ films.

## Abstract (web summary)

While water-splitting electrocatalysts enable energy storage in carbon-neutral fuels, a recent challenge has been the discovery and understanding of catalyst active sites. Here, authors find domain boundaries in MoS_2_ materials to present high-activity, stable, and scalable sites for H_2_ evolution.

## Introduction

Hydrogen evolution reaction (HER) process is crucial to the production of hydrogen, the most efficient and environmental-friendly fuel. Platinum (Pt) and Pt-based materials are known as the best electrocatalysts for HER so far, but they are also very scarce and expensive^1^. Recently, molybdenum disulfide (MoS_2_) has emerged as an active, earth-abundant, and inexpensive alternative to Pt and Pt-based electrocatalysts^2–7^. It is generally believed that the catalytic activity of MoS_2_ originates from its edges while its basal plane is rather inert, which limits the practical application of this material for HER^8–11^. In order to overcome the limited catalytic activity of the MoS_2_ basal plane, various techniques have been developed, such as phase engineering^12–15^, interface electronic coupling^16^, introducing active unsaturated defects^14^ and strain^17^. These techniques could improve the restricted factors (poor conductivity and limited active sites) for the potential of MoS_2_ in HER^18–20^. Recently, a pioneer strategy has been proposed by introducing S vacancies into the basal plane^17,21–25^, where gap states around the Fermi level allow hydrogen to bind directly to exposed Mo atoms. Considering the presence of dangling bonds in vacancy defects, these vacancy defects in MoS_2_ are easy to be poisoned and would lead to the surface instability from a HER point of view. To fully exploit MoS_2_ materials in realistic application, searching for alter

Herein, we report a facile route toward the activation of the monolayer MoS_2_ basal plane for HER by introducing domain boundaries, including both 2H–2H domain boundaries and 2H–1T-phase boundaries. We found that the domain boundaries can provide ultrahigh-density active sites, while still maintain the surface stability. Utilizing a multi-hierarchy design of these two types of boundaries, we are able to achieve a high basal-plane electrocatalytic performance with an exchange current density of 0.57 × 10^−4^ A cm^−2^, a Tafel slope of 73 mV dec^−1^, and a remarkable long-term operation stability over 200 h. We also demonstrate that such catalysts are scalable, e.g., over 4-inch wafer scale, pushing a crucial technological step toward practical applications.

## Results

### 2H–2H domain boundaries and 2H–1T-phase boundaries for HER

**Fig. 1:**
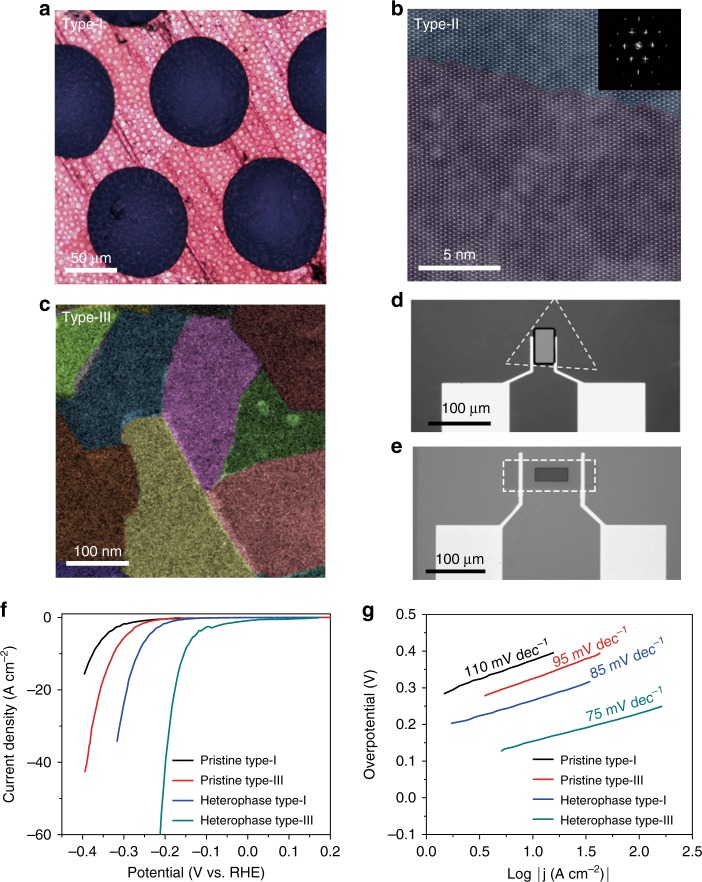
Activity of 2H–2H domain boundaries and 2H–1T-phase boundaries for HER. **a** Optical image of the type-I MoS_2_ with individual domain size of few hundreds microns transferred onto a TEM grid. **b** HRTEM images of as-grown type-II MoS_2_ film with highly orientated domains of few microns for the domain sizes. **c** False-color dark-field TEM image of type-III MoS_2_ with high-density 2H–2H domain boundaries. **d**, **e** Optical microscope image of a individual MoS_2_ single-crystal domain without 2H–2H domain boundaries (**d**) and polycrystalline ML–MoS_2_ with domain boundaries (**e**). Dashed regions indicate the HER window opened on the basal plane. **f** Polarization curves of the pristine type-I MoS_2_ (without any domain boundaries), pristine type-III MoS_2_ (with 2H–2H domain boundaries), heterophase type-I MoS_2_ (with 2H–1T-phase domain boundaries), and heterophase type-III MoS_2_ (with both 2H–2H and 2H–1T domain boundaries), respectively. **g** Tafel plots of the corresponding curves in **f**

In this study, we investigated both single-crystalline and polycrystalline 2H-phase monolayer MoS_2_ samples grown by chemical vapor deposition (CVD) for HER. The former samples (type-I) were grown on sapphire substrates with individual domain size of a few hundred micrometers (refer to ref. ^25^ for our previous work)^26^. The latter samples were continuous film samples grown either on sapphire substrates (type-II) with highly orientated domains of a few micrometers (refer to ref. ^26^ for our previous work)^27^ or on SiO_2_/Si substrates (type-III) with randomly orientated domains of a few hundred nanometers (refer to ref. ^27^ for our previous work)^28^. Figure 1a–c show typical optical and false-color transmission electron microscope (TEM) images of these different monolayer MoS_2_ samples to illustrate their domain sizes and the 2H–2H domain boundaries. Hence, we have three types of MoS_2_ samples for comparable investigation of the HER performances: type-I free of 2H–2H domain boundaries, type-II with low density of 2H–2H domain boundaries, and type-III with high density of 2H–2H domain boundaries (further details in Supplementary Fig. 1). Note that the 2H–2H domain boundaries in our MoS_2_ samples are not perfectly straight. These boundaries usually consist of various configurations, typically including arrays of 4–6 rings (4|6), 6–8 rings (6|8), 5–7 rings (5|7), and 4–4 rings (4|4) as shown in Supplementary Fig. 2. The high-resolution TEM and X-ray photoelectron spectroscopy (XPS) were used to confirm that all samples are of high quality with low-defect density and very clean surfaces even after transfer processes (Supplementary Fig. 3). Hence, the influence of defects in different types of samples can be excluded.

In order to investigate whether the 2H–2H domain boundaries could serve as active sites in HER, local probe characterizations were first performed (further details in Supplementary Note 1 and Supplementary Fig. 4). Figure 1d and e show two typical HER devices fabricated from type-I and type-III samples, respectively. Note that these devices were protected by polymethyl methacrylate (PMMA) masks with only small windows exposed on the MoS_2_ samples in the center to avoid the contribution from the MoS_2_ edges during the HER characterizations. The corresponding HER polarization curves and Tafel plots of both devices are shown in Fig. 1f, g, respectively. It can be clearly seen that type-III samples have better catalytic performances than type-I samples as evidenced by the drop of the overpotential from ~ 375 mV to ~ 325 mV at current density of 10 A cm^−2^ and the Tafel slope from ~ 110 to ~ 95 mV dec^−1^. Since the qualities of the two samples are very similar, except for the density of domain boundaries, we thus can draw a conclusion that the enhanced HER activity in type-III samples comes from the 2H–2H domain boundaries.

Then we explored the possibility of further introducing 2H–1T-phase boundaries as active sites in HER. In order to produce such boundaries, we performed post low-energy Ar–plasma bombardments on pristine type-I and type-III samples to induce the 2H-to-1T-phase transition (refer to ref. ^28^ for our previous work)^29^. Note that this phase transition is quite local and the resulted samples consist of a mosaic texture of nearly half 2H- and half 1T-phases, exhibiting an average domain size of a few nanometers and high-density phase boundaries (Supplementary Fig. 5). HER performance of two typical heterophase devices similar to those described above is shown in Fig. 1f, g. For type-I sample after phase transition, a drop of the overpotential from ~ 370 to ~ 260 mV at current density of 10 A cm^−2^ and the Tafel slope from ~ 110 to ~ 85 mV dec^−1^ can be clearly seen, suggesting that 2H–1T-phase boundaries are more efficient for HER as active reaction sites than 2H–2H boundaries. Besides, the metallic 1 T phase offers better charge transport capability than 2 H phase, which is consistent with previous report^16^. Notably, the heterophase type-III sample exhibits the lowest overpotential of 200 mV and Tafel slope of 75 mV dec^−1^. In order to confirm that no other defects in the heterophase structure contributed prominently to the enhanced HER activity, we performed atomic force microscopy (AFM) and STM characterizations. Only few S vacancies (~1.9%) having little effect on HER can be found without any other defects in the heterophase sample (details in Supplementary Figs. 6–7 and Supplementary Note 2)^17,25,30^. Particularly, we also found that these S vacancies would not introduce gap states that allow favorable hydrogen adsorption (Supplementary Fig. 7). We have also performed thermal annealing for our phase-changed samples to investigate the effect of phase boundaries. Thermal annealing was carried out in vacuum at 600 °C for 1 h. This process can recover from all 1 T phases back to 2 H phases in our sample (as confirmed in Supplementary Fig. 8a). Meanwhile, after annealing treatment, the HER performance degrades to the same level of pristine MoS_2_, as shown in Supplementary Fig. 8b. On the basis of these results, we concluded that the phase boundaries are dominant active sites in our heterophase MoS_2_ samples while those S vacancies contribute less to enhance the HER performance. Thus the composite structure containing both high-density domain and phase boundaries is the most promising candidate for HER.

### Mechanism of the activation of the MoS_2_ basal plane by boundaries

**Fig. 2:**
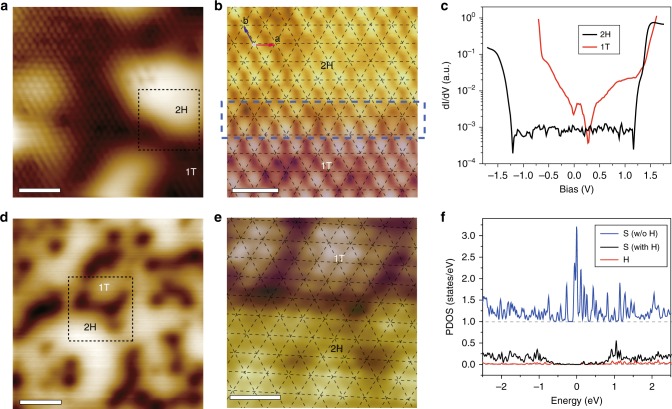
Hydrogen adsorption at 2H–1T-phase boundaries. **a** STM topography of as-treated MoS_2_ showing mixed 2 H (bright) and 1 T (dark) domains. **b** Zoom-in image of a domain boundary as denoted by a dashed square in **a**, where 2H and 1T phases are rendered with yellow and purple colors, respectively. The lattice grid of 2H phase is superimposed on the image to highlight the lateral sliding of S atoms in 1T phase. The black arrows indicate the sliding direction [120]. The two in-plane primitive vectors are shown in the upper left of **b**. **c** dI/dV spectra taken on the 2H and 1T phases, suggesting the semiconducting 2H phase with a 2.5-eV bandgap and metallic 1T phase. **d** STM image of the MoS_2_ after hydrogenation, showing prominent depression features. The sample bias was chosen such that the apparent heights of 2 H and 1 T phases become similar, to highlight the depression features. **e** Zoom-in image of the depression features as denoted by a dashed square in **d**, indicating that the depression features are located exactly at the boundary of 2H and 1T phases. Set points of the STM images: **a**: 1.5 V, 50pA; **b**: 1 V, 10 pA; **d**: 2 V, 10 pA; **e**: 0.5 V, 50pA. Set point of dI/dV spectra:1.5 V, 50 pA **c**. **f** Projected density of states (PDOS) of S and H atoms at the phase boundary before and after hydrogenation. Blue curve: PDOS of S atoms before hydrogenation, black curve: PDOS of S atoms with H bonded, red curve: PDOS of absorbed H atoms. Scale bar: 2 nm **a**, 0.7 nm **b**, 1.5 nm **d**, 0.7 nm **e**

In order to confirm the role of 2H–1T-phase boundaries in the basal plane of MoS_2_ in HER, we performed scanning tunneling microscopy (STM) and scanning tunneling spectroscopy (STS) characterizations. Since STM/STS measurements require conductive substrates, MoS_2_ samples were epitaxially grown on graphite by CVD and treated by Ar–plasma to induce phase transitions subsequently. Figure 2a shows a typical topographic STM image of the monolayer heterophase MoS_2_, where bright and dark regions correspond to the 2 H and 1 T phases, respectively. A zoom-in image at one-phase boundary is shown in Fig. 2b with the top sulfur (S) atoms clearly resolved. The sliding of S atoms in 1 T phase with respect to the 2 H phase further confirmed coexistence of the two phases (details in Supplementary Fig. 9). As shown in Fig. 2c, 2H-phase domains have bandgaps as usual, e.g., ~2.5 eV; in contrast, 1T-phase domains are metallic without bandgaps.

In HER process, stable hydrogen adsorption at active sites is a crucial step. Therefore, we simulated this step via hydrogenating the heterophase sample surface by the atomic hydrogen (see Supporting Information for more details). After hydrogenation, additional depression features with atomic-size width appear exactly at the phase boundaries (Fig. 2d, e), suggesting that the atomic hydrogen prefers to adsorb at the S sites of phase boundary. Density functional theory (DFT) calculations were also carried out to confirm the STM observations. Simulated results indicate that the formation of S–H covalent bonds can lead to a significant reduction of the density of states (DOS) of S atoms around the Fermi level (Fig. 2f), which is consistent with the apparent depression observed in the STM images due to the adsorption of H atoms on the S atoms.

**Fig. 3:**
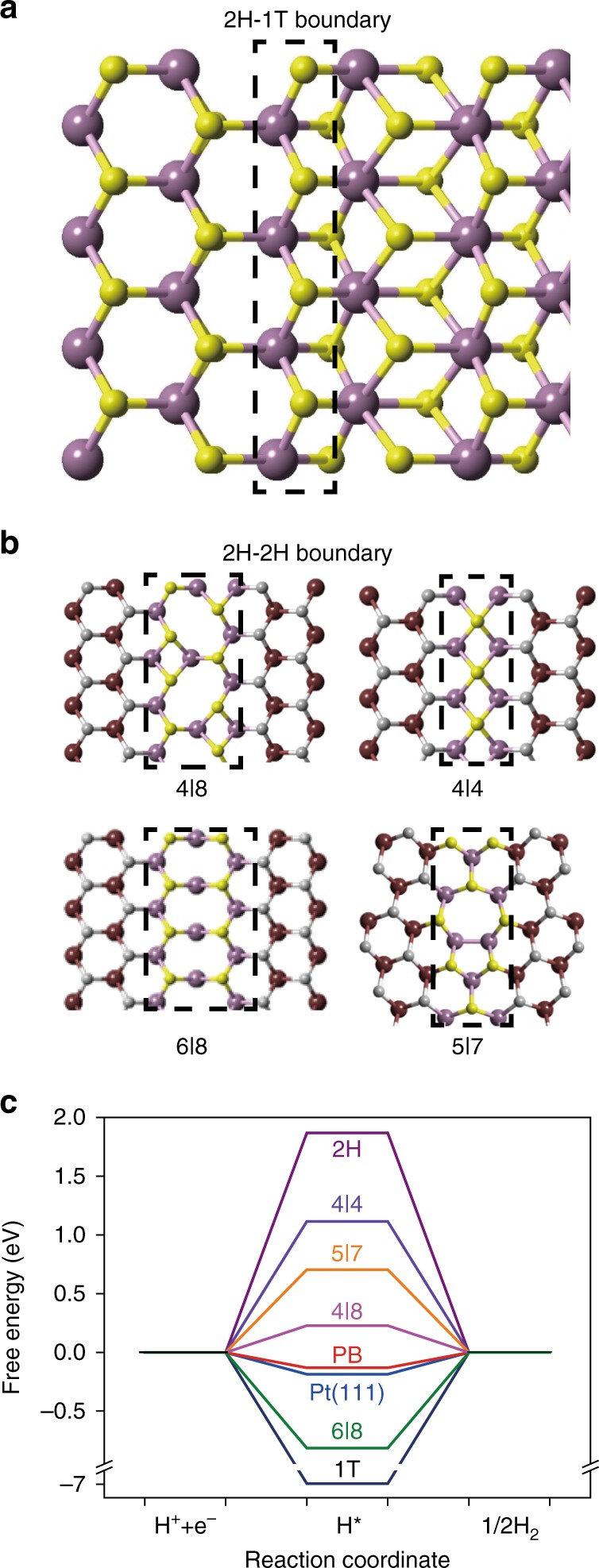
Theoretical simulations. **a**, **b** The top views of the atomic model of the zigzag type 2H–1T-phase boundaries and four kinds of 2H–2H boundaries (4|8, 6|8, 4|4, 5|7). **c** A comparison of the Gibbs free energies of the adsorbed H on 2H-phase of MoS_2_, 1T-phase of MoS_2_, Pt(111) surface, 2H–1T-phase boundaries (PBs) and four kinds of 2H–2H boundaries in the context of HER

Next, we performed further DFT calculations on the 2H–2H grain boundaries and the 2H–1T-phase boundaries to investigate their catalytic activities (see Supporting Information for computational details). Figure 3a, b show the structural model of 2H–1T-phase boundaries and four typical kinds of 2H–2H boundary configurations (4|8, 6|8, 4|4, 5|7)^24^. Figure 3c shows the calculated Gibbs free energy of the adsorbed atomic hydrogen (ΔG_H*_). Note that ΔG_H*_ is a widely accepted indicator for the catalytic activity and the optimal value is ΔG_H* _= 0 eV, where hydrogen is bounded neither too strongly nor too weakly^31^. For comparison, we also performed calculations on Pt (111) surface, basal plane of 2H–MoS_2_ and 1T–MoS_2_ with the same sized supercells as for the phase boundaries, yielding ΔG_H* _= −0.18 eV, 1.87 eV, and −6.97 eV, respectively (Fig. 3b). These numbers are consistent with previous calculations^10,32–36^. Due to the high instability of the pristine 1 T phase, the initial binding of hydrogen on a pure 1T-phase basal plane is quite strong, which results in heavy relaxation of the adsorption area. This releases most of the 1 T energy, making the structure rather inert for the further adsorption of hydrogen atoms^14,37,38^. Moreover, for the 1 T phase confined within 2 H phase by interfaces, the strong relaxations at the interfaces have the similar effect as the initial H adsorption, which prevents the confined 1 T phase to adsorb more H atoms favorably (further details in Supplementary Fig. 10). This can explain the absence of H adsorption at the nanometer-size 1 T phase as shown in the STM image (Fig. 2d, e). The Gibbs free energy of all the H adsorption sites in 2H–2H domain boundaries are much smaller than that of the perfect MoS_2_ basal plane (1.87 eV), indicating that 2H–2H boundaries can indeed break the inertia of the basal plane and enhance the interaction between the H atom and the adsorption sites. Impressively, the 2H–1T-phase boundaries exhibit ΔG_H*_ = −0.13 eV (Fig. 3b), very close to that of the Pt (111) surface and Mo-edge of 2H–MoS_2_. Thus, phase boundaries in the basal plane of monolayer MoS_2_ could serve as effective sites to tune hydrogen reaction barriers and optimize the overall kinetics of H_2_ evolution^39,40^.

### Multi-hierarchy monolayer MoS_2_ catalysts for HER

**Fig. 4:**
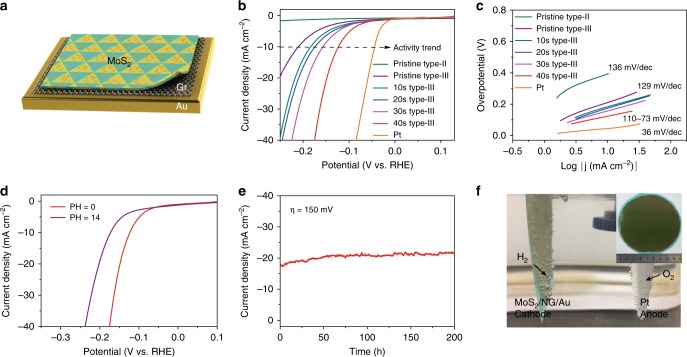
Multi-hierarchy monolayer MoS_2_ catalysts for HER. **a** Schematic structure of the multi-hierarchy MoS_2_ catalysts with both high density of domain and phase boundaries. **b** Polarization curves for pristine type-II samples, pristine type-III samples, a series of heterophase type-III samples with different phase boundary densities and Pt. **c** Tafel plots of the corresponding curves in **b**. **d** HER performance of a multi-hierarchy MoS_2_ catalyst in 0.5-M H_2_SO_4_ (red curve) and 1-M KOH (purple curve). **e** Time-dependent current density curve for a multi-hierarchy MoS_2_ catalyst under static overpotential of 150 mV for 200 h. **f** Demonstration of the catalytic HER activity in 0.5 M H_2_SO_4_ from a multi-hierarchy MoS_2_ catalyst with a size of 4 inches in diameter. Inset: photograph of pristine as-grown wafer-scale MoS_2_ on sapphire substrate

Based on the above experimental and theoretical results, we can conclude that both domain boundaries and phase boundaries can serve as active sites in HER. More boundaries, in principle, should offer better HER performance. We thus investigated the effect of boundary density in a systematic way. MoS_2_ electrodes for electrocatalytic HER testing were fabricated from pristine type-II samples (with low density of 2H–2H domain boundaries), pristine type-III samples (with high density of 2H–2H domain boundaries), and a series of heterophase type-III samples with varying density of 2H–1T-phase boundaries. As mentioned above, the heterophase MoS_2_ can be produced by Ar–plasma bombardments; while the percentage of 1T-phase in 2H-phase matrix, thus the density of phase boundaries, can be actually tuned by the treatment durations. The illustrated multi-hierarchy catalysts for HER characterizations is shown in Fig. 4a. ML–MoS_2_ catalysts were supported on graphene (Gr)/Au films (structure and fabrication details discussed in Supplementary Note 1 and Supplementary Figs. 11–13). Note that the underlying graphene layers, as an internal electron transport channels, can decrease the resistive loss and accelerate electron transport from the MoS_2_ film to electrodes^11^. Polarization curves (Fig. 4b) and Tafel plots (Fig. 4c) of these samples were measured in 0.5 M sulfuric acid electrolyte using a standard three-electrodes configuration (see the Methods section for more details). Note that, the overpotential at 10 mA/cm^2^ decreased linearly with increasing the phase-boundary density of MoS_2_ tuned by the treatment durations, which confirmed the effect of phase boundaries in HER (further details see Supplementary Figs. 14, 15).

The control sample is a commercial Pt foil, which exhibits a nearly zero overpotential. From these data shown in Fig. 4b, we can clearly see that samples with either more domain boundaries or more phase boundaries can yield better HER performance in terms of reduced overpotentials and Tafel slopes. The best structure is a composite with both high-density domain and phase boundaries, as shown in the illustrated drawing of Fig. 4a. Notably, the best sample presents a very low onset potential of ~100 mV, a low overpotential of ~136 mV, a Tafel slope of ~73 mV dec^−1^, an extremely large cathodic current density of ~78 mA cm^−2^ at *η* = 200 mV, and an exchange current density of 57 μA cm^−2^, all of which are better than or comparable to sulfur vacancies (details see Supplementary Fig. 16) and previous results from the edge-dominated MoS_2_ catalysts^33,41,42^.

A remarkable feature of these composite boundary catalysts is that they can work in both acidic and alkaline conditions with similar performance, considering that only a few candidates could do this job^43^. Polarization curves (Fig. 4d) measured in 1 M KOH (pH = 14) present an overpotential of ~176 mV at 10 mA cm^−2^, which is slightly larger than that in acidic conditions. We also carried out stability testing, which is another important aspect in practical applications, for the multi-hierarchy MoS_2_ catalysts in Fig. 4e. Surprisingly, it can work for over 200 h without any noticeable degradation of current density. We also investigated these two type of boundaries, respectively, and both of them show excellent durability (see Supplementary Fig. 17). Thus we attribute this excellent durability to the stable structures of monolayer MoS_2_ with 2H–2H and 2H–1T boundaries. Such structures will not be degraded during the HER process, although they are just monolayers. Finally, the catalysts with composite boundary can also be easily scaled up. As a proof-of-concept demonstration, a 4-inch wafer-scale catalysts, exhibited in Fig. 4f, was prepared for HER. The wafer-scale catalysts sample still exhibit good HER properties (see Supplementary Fig. 18 and Supplementary Movie 1 for more details).

## Discussion

In conclusion, we have both experimentally and theoretically verified that 2H–2H and 2H–1T domain boundaries in basal plane of ML–MoS_2_ could act as new highly active and tunable catalytic sites for HER. Based on the observed phenomena, we then achieved multi-hierarchy ML–MoS_2_ electrocatalysts containing both types of domain boundaries for HER. These electrocatalysts not only show remarkable electrocatalytic performances with a small overpotential of ~0.1 V and large cathodic currents, but have long-term stability and universality in both acidic and alkaline conditions. Moreover, the ML–MoS_2_ electrocatalysts with composite boundaries can be easily scaled up. Our results provide a comprehensive understanding of the HER mechanism for the MoS_2_ basal plane, as well as a facile route to design high-performance electrocatalysts.

## Methods

### Growth of the monolayer 2H–MoS_2_ using CVD method

A three zone furnace was used for CVD growth of MoS_2_. SiO_2_ (300 nm)/P + + Si and highly oriented pyrolytic graphite (HOPG) served as substrates. Sulfur (S) (Alfa Aesar 99.9%) and molybdenum trioxide (MoO_3_) (Alfa Aesar 99.999%) were used as precursors and loaded in zone I and II, respectively. The distance between the two sources was 22 cm. The substrates were put in the third zone. The temperatures of MoO_3_, S, and substrates were 560, 120, and 780 °C, respectively. Each temperature zone was kept stable for 20 min before the growth. During the growth, argon was used as carrying gas at a flow rate of 130 sccm, and the vacuum pressure was kept at 0.67 Torr.

### Formation of heterophase MoS_2_

Phase transition of MoS_2_ was also performed in the home-made, remote plasma system reported in our previous work^29^. An inductively coupled plasma was generated by dispersing a 20 W RF power at the entrance of a 4-inch quartz-tube furnace. The pressure in the tube furnace was fixed at ≈0.69 Torr for phase transition by flowing argon at 100 sccm and vacuum pumping. The process was carried out for 10, 20, 30, 40 s at room temperature, respectively.

### Characterization details

The as-grown layer monolayer MoS_2_ samples were characterized by optical microscopy, AFM (Bruker Icon microscope, tapping mode), Raman spectroscopy (532 nm laser, Horiba Jobin Yvon LabRAM HR-Evolution Raman), X-ray photoelectron spectroscopy (Kratos Analytical Axis Ultra).

### Electrochemical characterizations

All of the electrochemical measurements were performed in a typical three-electrode system on electrochemical workstation (Autolab PGSTAT 302 N). A Pt foil or a glassy carbon electrode (for long time test) were used as counter electrodes, and saturated Ag/AgCl electrode serve as the reference electrode. The linear sweep voltammetry (LSV) with the scan rate of 5 mV s^−1^ was carried out. All the applied potentials were converted to reversible hydrogen electrode (RHE) potentials scaled using the equation E (vs. RHE) = E (vs. Ag/AgCl) + 0.204 V + 0.0591 VPH, after IR correction. The stability tests for the heterophase MoS_2_ was performed using chronoamperometry at a constant applied overpotential.

### Computational details

First-principles calculations based on DFT were carried out by using the Vienna Ab initio Simulation Package (VASP)^7,10^. The interactions between valence electrons and ions were treated with the projector-augmented wave (PAW) method^8^. The exchange-correlation interactions were described by generalized gradient approximation (GGA) with the Perdew−Burke−Ernzerhof (PBE) functional^11^. The electron wave functions were expanded in a plane-wave basis set with cutoff energy of 520 eV. The convergence criterion for residual force on each atom during structure relaxation was set to 0.02 eV/Å, and the geometries were relaxed to minimize the total energy of the system until a precision of 10^–4^ eV was reached.

## Supplementary information

Supplementary Information

Description of Additional Supplementary Files

Supplementary Movie 1

Source Data

## Data Availability

All data are available from the authors upon reasonable request. All source data underlying Figs. 1a, f, 2c, f, 3c, 4b–e and Supplementary Figs. 1g, h, 3d, e, 5a–c, 7c, 8a, 12a, d, 13a–c, 16, 17 and 18d are provided as a Source Data file.
