# Supplementary material for: Boundary activated hydrogen evolution reaction on monolayer MoS2
Source: Nat Commun. 2019 Mar 22;10:1348. doi: 10.1038/s41467-019-09269-9 (PMC6430794; doi:10.1038/s41467-019-09269-9)
Supplement: Supplementary file 1 — Supplementary Information [file 41467_2019_9269_MOESM1_ESM.pdf]

Supplementary Materials for

Boundary activated hydrogen evolution reaction on monolayer MoS_2

Jianqi Zhu[1,5†], Zhi-Chang Wang[3†], Huijia Dai[4†], Qinqin Wang[1,2], Rong Yang[1,2], Hua Yu[1] , Mengzhou Liao[1,2], Jing Zhang[1], Wei Chen[1], Zheng Wei[1,2], Na Li[1,2], Luojun Du[1], Dongxia Shi[1,2], Wenlong Wang[1,2], Lixin Zhang[4*], Ying Jiang[3,6,7*] and Guangyu Zhang[1,2,6,8*]*

[1]*CAS Key Laboratory of Nanoscale Physics and Devices, Institute of Physics, Chinese Academy of Sciences, Beijing 100190, China*

[2]*School of Physical Sciences, University of Chinese Academy of Sciences, Beijing 100190, China*

[3]*International Center for Quantum Materials, School of Physics, Peking University, Beijing 100871, China*

[4]*School of Physics, Nankai University, Tianjin 300071, China*

[5]*School of Physics and Electronic Engineering , Sichuan Normal University, Chengdu, Sichuan 610101, China*

[6]*Collaborative Innovation Center of Quantum Matter, Beijing 100190, China*

[7]*CAS Center for Excellence in Topological Quantum Computation, University of Chinese Academy of Sciences, Beijing 100190, P. R. China*

[8]*Beijing Key Laboratory for Nanomaterials and Nanodevices, Beijing 100190, China*

[†]*Authors contributed equally to this work.*

[*]*Corresponding authors. E-mail: gyzhang@iphy.ac.cn; yjiang@pku.edu.cn;* lxzhang@nankai.edu.cn*; ryang@iphy.ac.cn*

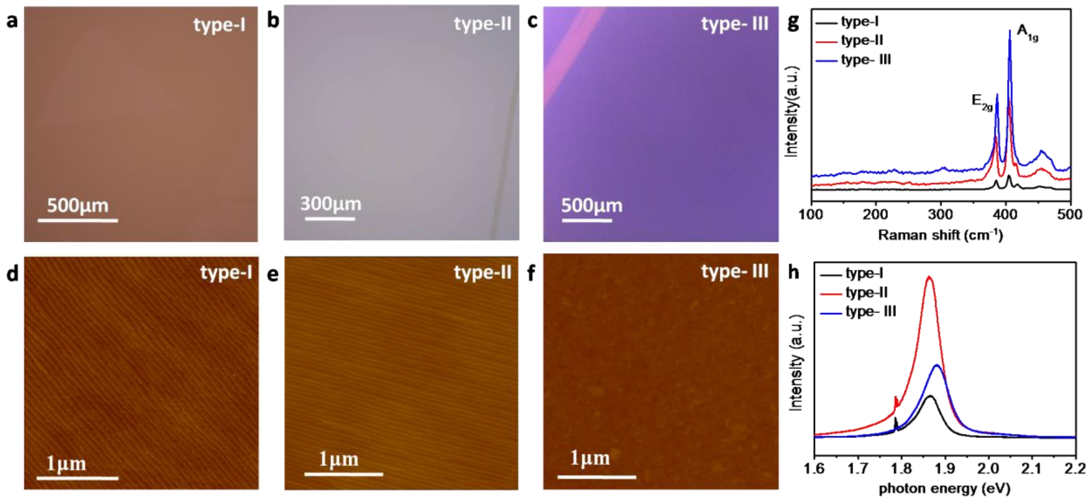

Supplementary Figure 1 | Different types of As-grown monolayer MoS_2 samples. a-c, Optical image of the (**a**) type-I, (**b**) type-II and (**c**) type-III MoS_2. **d-f**, The atomic force microscopy images of the corresponding as-grown MoS_2 samples in (**a-c**). **g,h**, (**g**) Raman spectra and (**h**) PL spectraof these three types of samples.

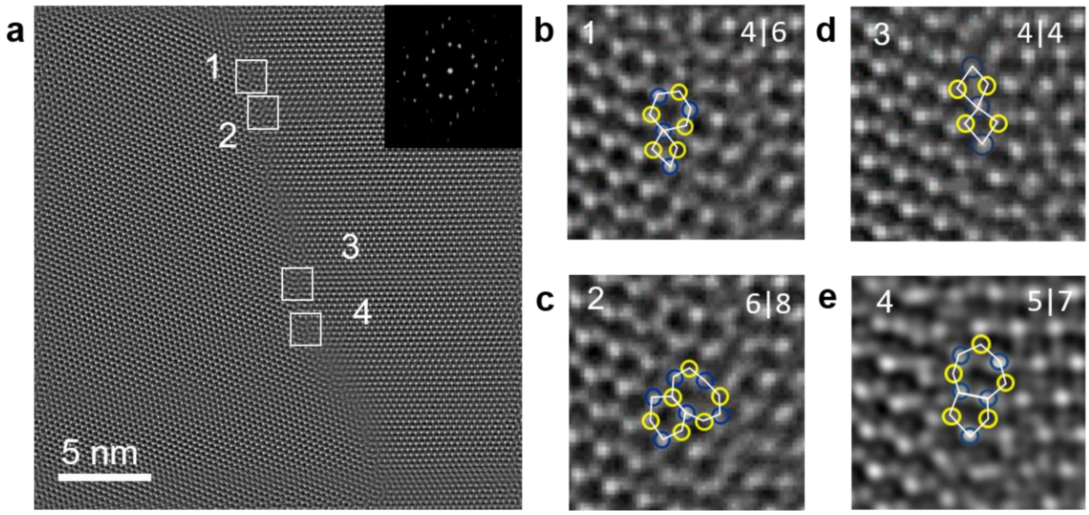

Supplementary Figure 2 | Scanning transmission electron microscope characterizations of 2H-2H boundaries. a, STEM image of 23° 2H-2H boundaries in as-grown monolayer MoS_2 containing different configurations including arrays. **b**-**e**, High resolution STEM images of (**b**) 4-6 rings (4|6), (**c**) 6-8 rings (6|8), (**d**) 5-7 rings (5|7), (**e**) 4-4 rings (4|4).

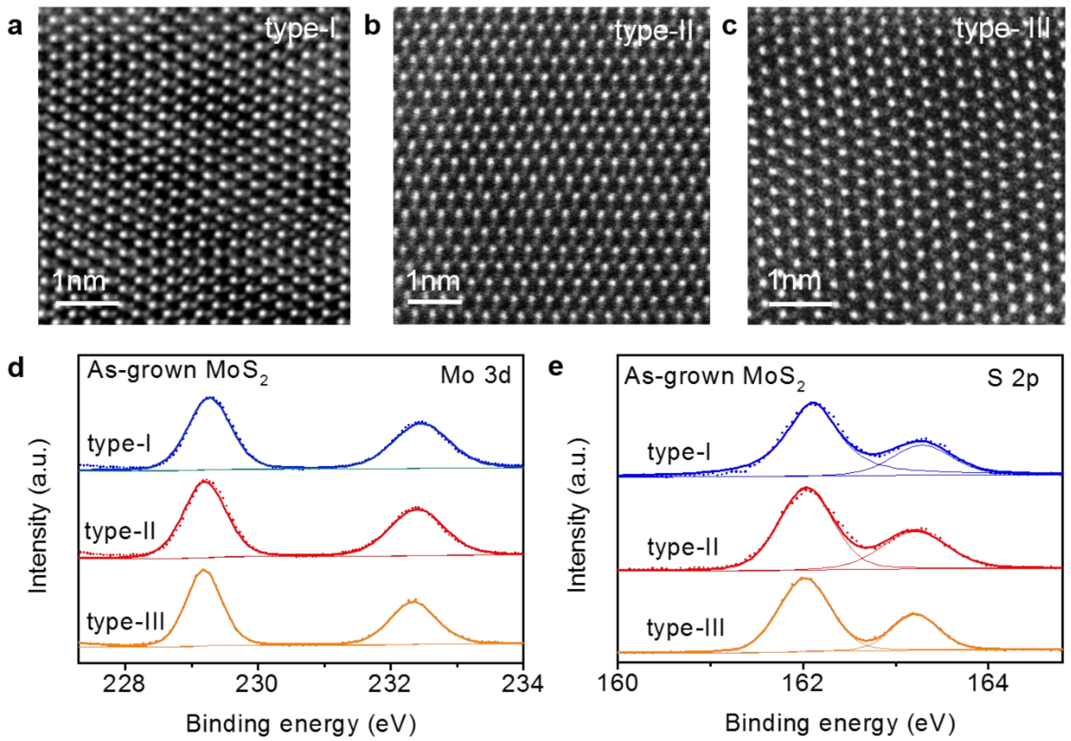

Supplementary Figure 3 | Characterizations of monolayer MoS_2. a-c, Atomic resolution ADF-STEM image of as-grown (**a**) type-I, (**b**) type- II, (**c**) type- III samples. **d**,**e**, XPS (**d**) Mo 3d and (**e**) S 2p peaks of different type of as-grown MoS_2 samples.

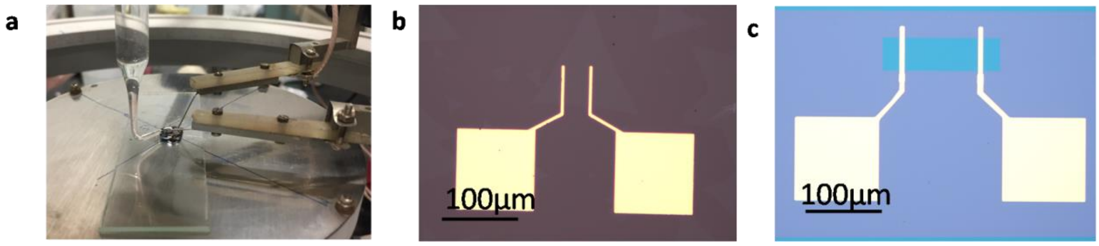

Supplementary Figure 4 | Electrochemical set-up for the local HER measurement. **a,** Photograph of the electrochemical microcell **b,c**, The gold electrodes are fabricated to contact (**b**) type-I, (**c**) type- III samples.

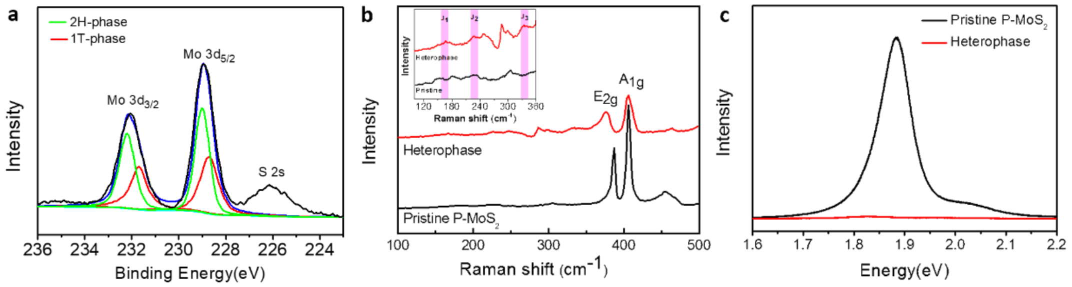

Supplementary Figure 5 | Characterizations of phase-changed monolayer MoS_2. **a**, XPS spectra showing Mo 3d and S 2s core level peak regions for the pristine and plasma-treated MoS_2 **b**, Raman spectra of ML-MoS_2 and plasma-treated MoS_2. New peaks can be observed in the plasma-treated monolayer MoS_2 confirming a mixture of 1T and 2H phase MoS_2. The inset shows enlarged spectra of new characteristic Raman peaks for the 1T phase MoS_2. **c**, PL spectra of plasma-treated Monolayer MoS_2.

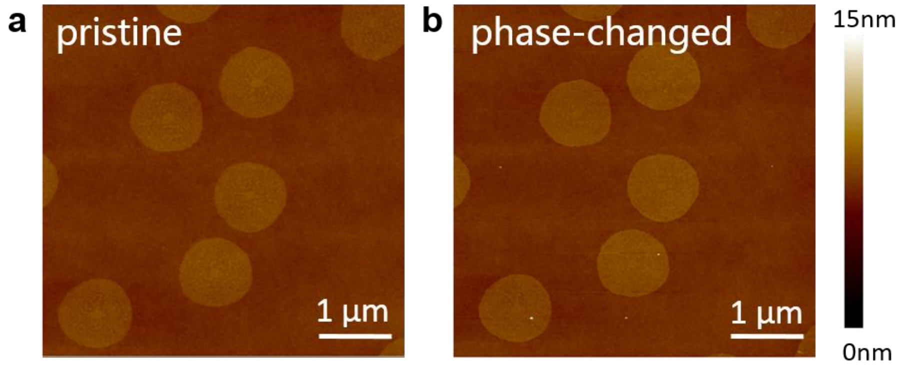

Supplementary Figure 6 | Atomic force microscopy characterizations of monolayer MoS_2 before and after phase transition. a,**b** AFM image of monolayer MoS_2 on Si/SiO_2 substrate (**a**) before and (**b**) after Ar-Plasma treatment.

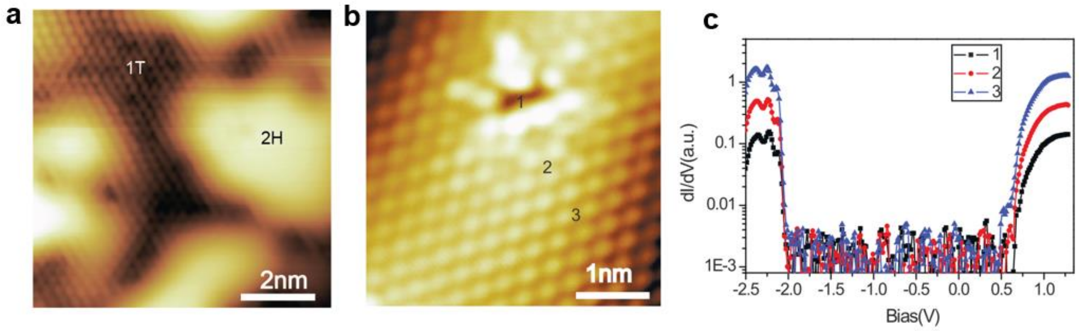

Supplementary Figure 7 | Scanning tunneling microscopy characterizations of monolayer MoS_2. a,b, STM image of the coexistence phase (**a**) and defects in the 2H phase of the coexistence phase (**b**). **c**, STS around the defect. Set points: 1.5V, 50pA(**a**); 0.5V,20pA (**b**); 1V,50pA(**c**).

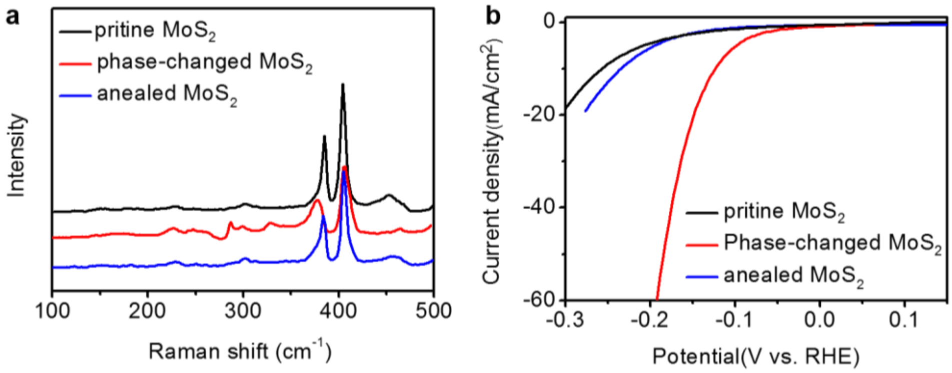

Supplementary Figure 8 | Raman characterizations of phase-changed MoS_2. a, Raman spectra of as-grown MoS_2, phase-changed MoS_2 and annealed phase-changed MoS_2. **b**, HER performances of as-grown MoS_2, phase-changed MoS_2 and annealed phase-changed-MoS_2.

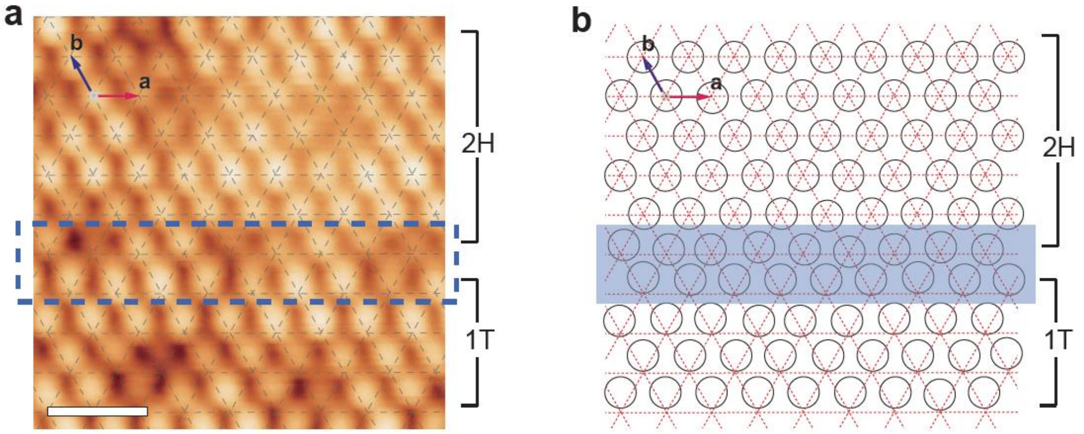

Supplementary Figure 9 | The sliding of S atoms in 1T phase. a, STM atomic resolution image of the phase boundary. **b**, In order to eliminate the influence of background STM image on the visualization of sliding of S atoms, the STM image is removed. The grids correspond to the lattice of 2H phase, while the circles correspond to the S atoms in 1T and 2H phases.

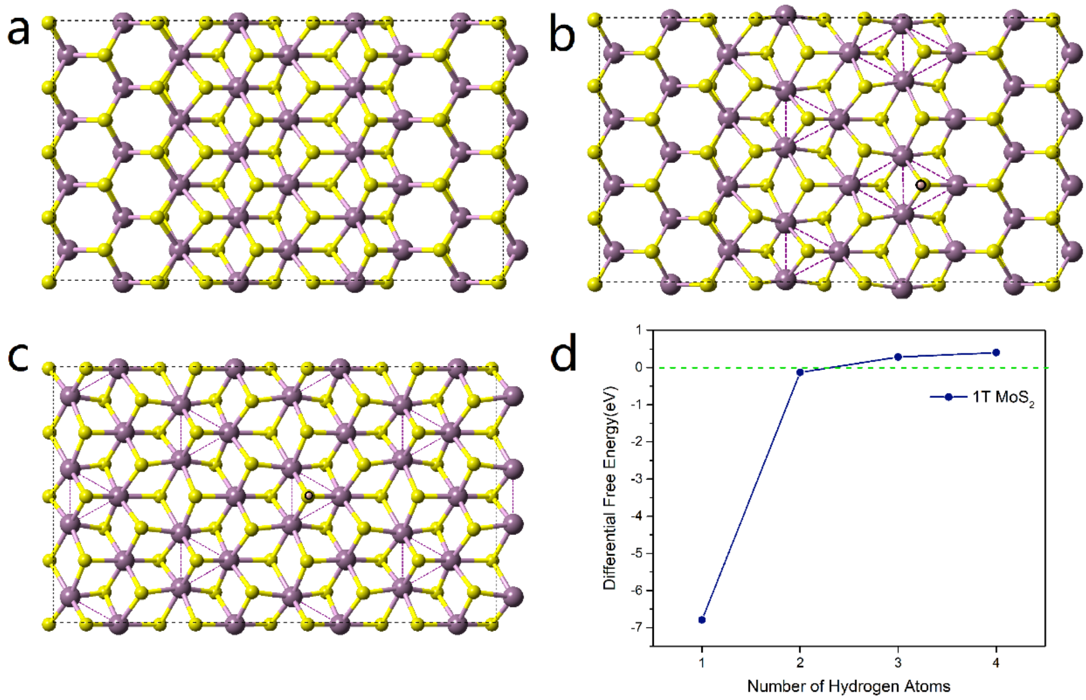

Supplementary Figure 10 | Hydrogenating the heterophase sample surface by the atomic hydrogen. a, The structure of heterophase MoS_2 with phase boundaries. **b**, Distorted structure of (**a**) heterophase MoS_2 after one hydrogen (dark circle) adsorbed on S atoms. **c**, Relaxation of pristine 1T phase structure after one hydrogen atom adsorbed on the basal plane. **d**, The differential free energy of hydrogen adsorption as a function of hydrogen atom's number on pristine 1T-MoS_2 surface.

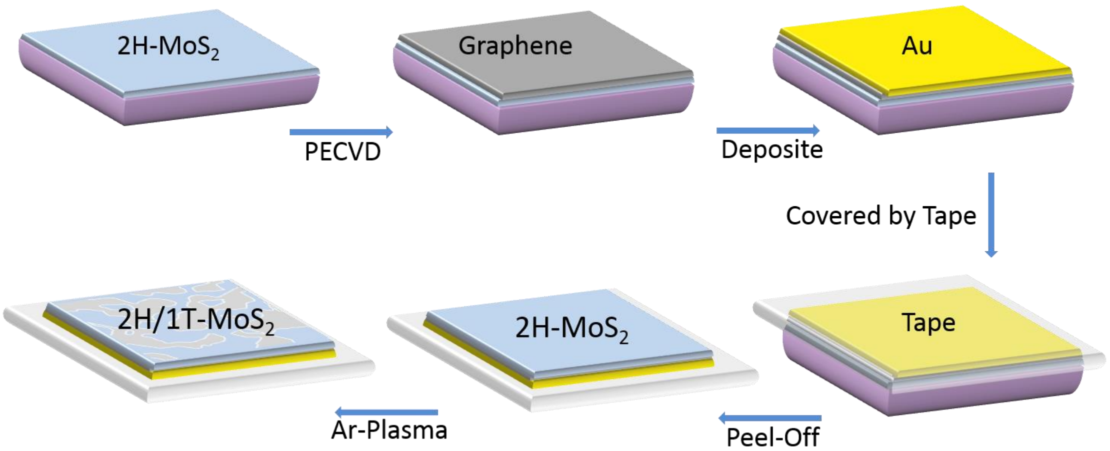

Supplementary Figure 11 | Fabrication process of MoS_2 multi-hierarchy catalysts. Schematic depiction of the heterophase-MoS_2/ graphene /Au electrode fabrication process.

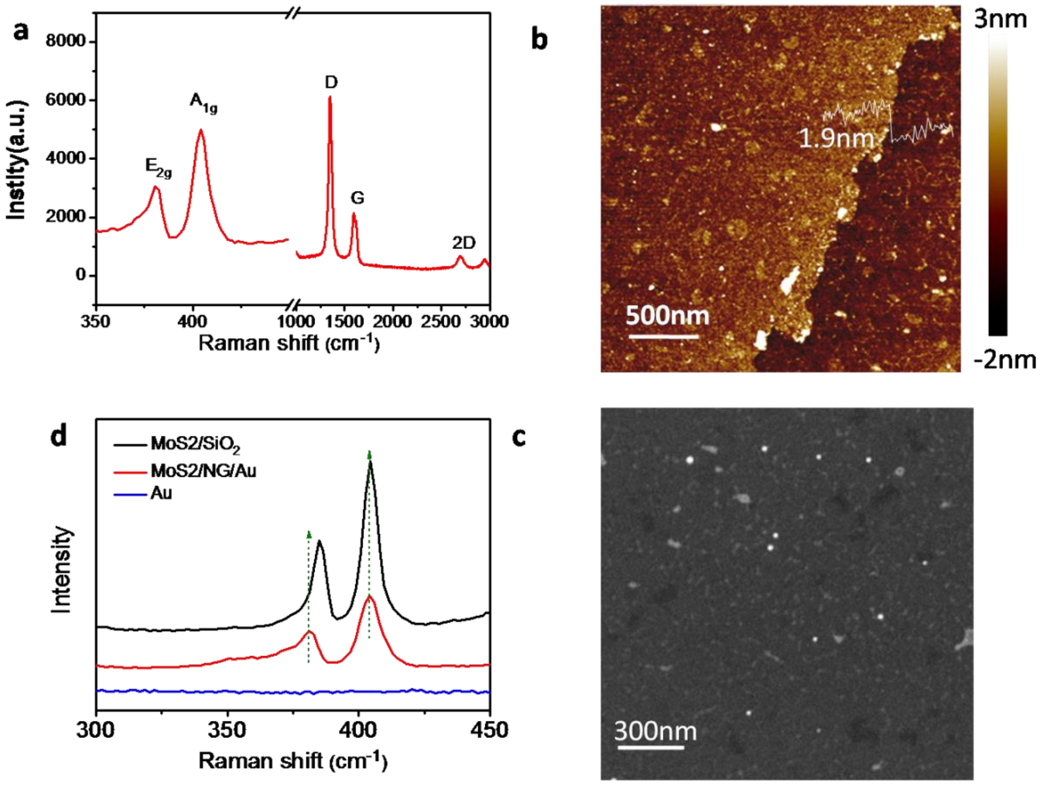

Supplementary Figure 12 | Characterizations of MoS_2 multi-hierarchy catalysts. **a,** Raman spectrum of the as-grown MoS_2/ nanographene films. **b**, The atomic force microscopy image of the as-grown MoS_2/graphene films. **c**, SEM image of the as-grown MoS_2/graphene films. **d**, Raman spectrum of the pristine as-grown MoS_2, as-grown MoS_2/graphene films on Au foil and only Au foil.

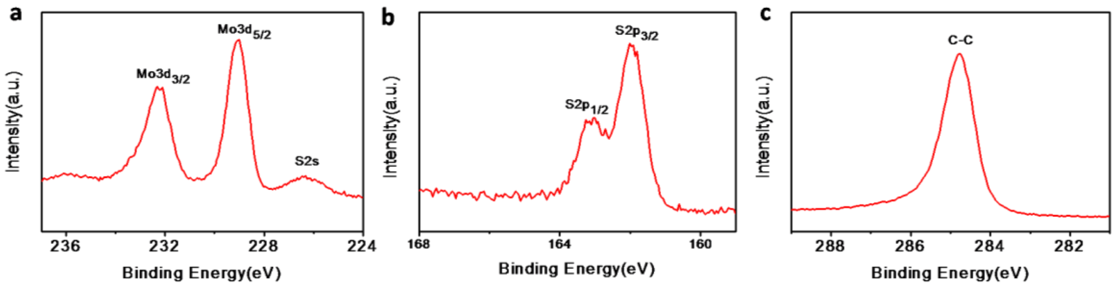

Supplementary Figure 13 | XPS characterizations of MoS_2 multi-hierarchy catalysts. XPS spectra of the as-grown MoS_2/graphene films: **a,** Mo 3d. **b,** S 2p. **c,** C 1s.

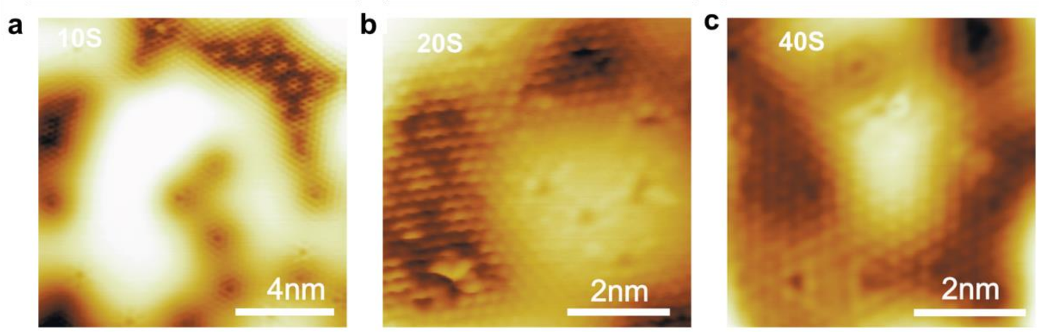

Supplementary Figure 14 | Typical STM images of different treating time monolayer MoS2 samples. **a**, 10S; **b**, 20S; **c**, 40S. Set points: 0.8V, (**a**) 20pA; 0.6V, (**b**) 5pA; 1.5V, (**c**) 50pA.

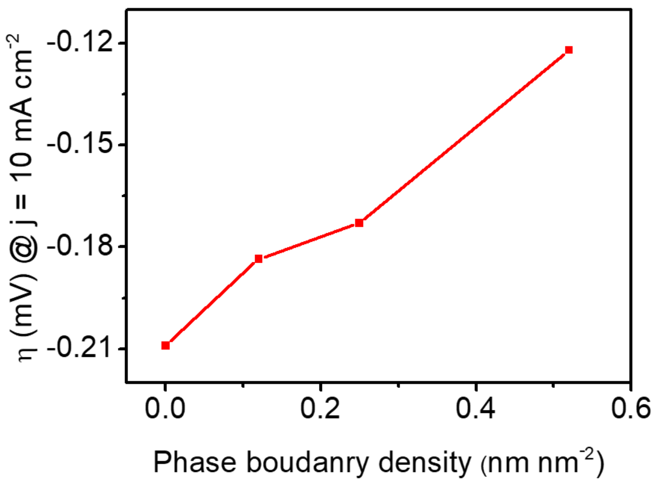

Supplementary Figure 15| The effect of phase boundaries. Statistical distributions of the overpotential at 10 mA/cm^2 as a function of phase boundaries density.

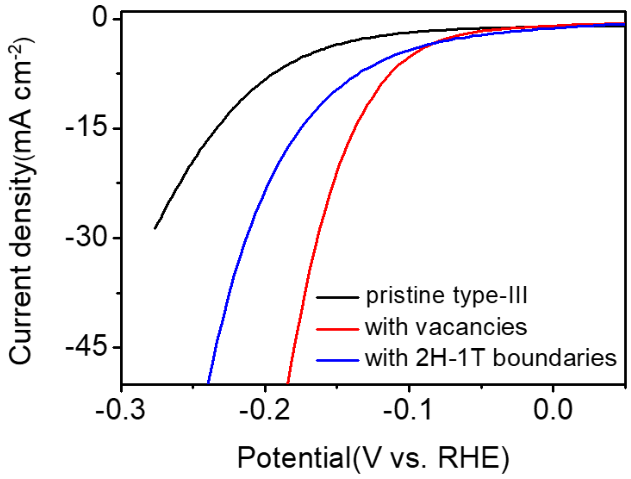

Supplementary Figure 16 | The effect of phase boundaries and sulphur vacancies. Polarization curves for pristine type-III MoS_2 (black), type-III MoS_2 with 19% Sulphur vacancies (blue) and type-III MoS_2 with 2H-1T boundaries (red).

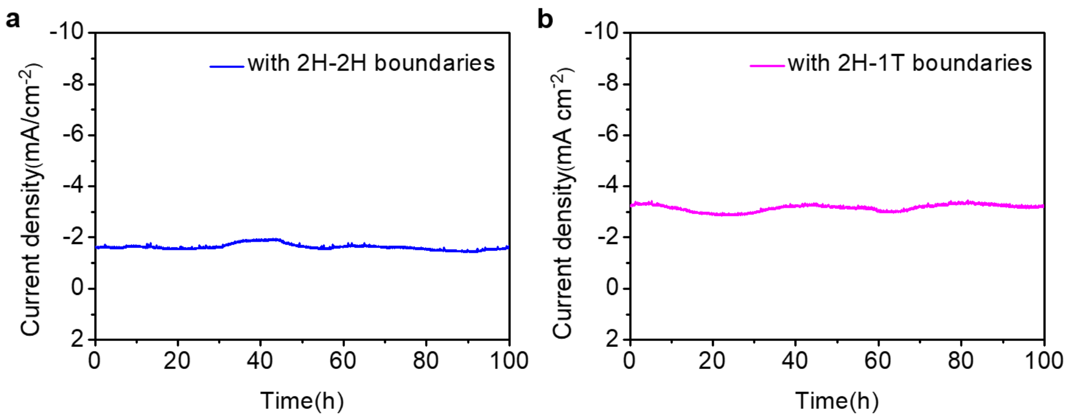

Supplementary Figure 17 | Durability of two type of boundaries. a,**b** Time-dependent current density curve for pristine (**a**) and phase-changed (**b**) type-II MoS_2 catalyst under static overpotential of 100 mV for 100 h.

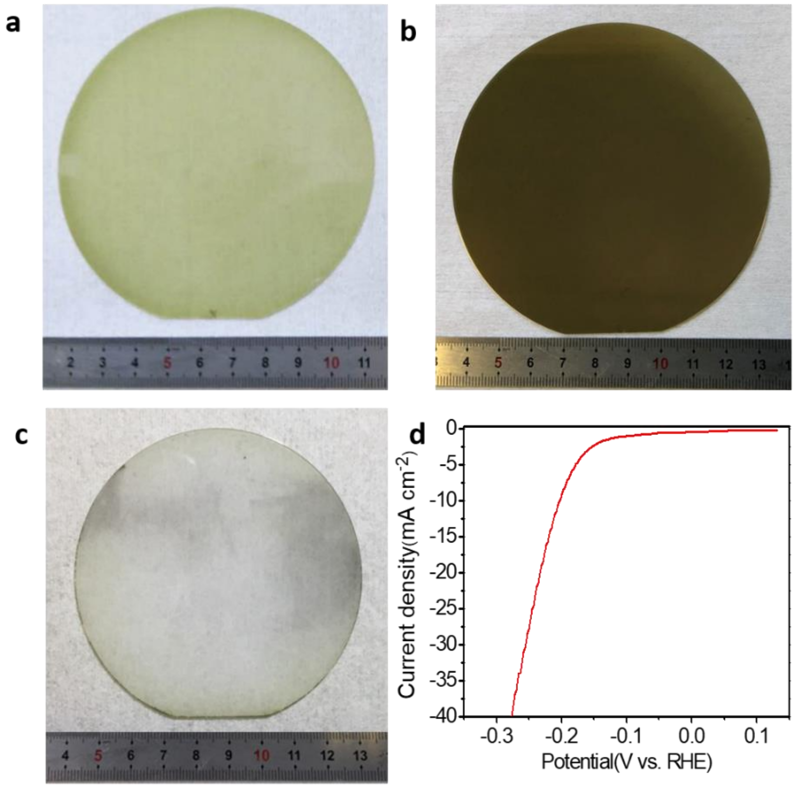

Supplementary Figure 18| The wafer-scale catalysts. a-c, Potograph of pristine as-grown wafer-scale MoS_2 on (**a**) sapphire substrate, as-grown MoS_2/nanographene sample deposited with (**b**) gold and sapphire substrate separated from (**c**) Au/Gr/MoS_2 films. **d**, Polarization curves for the 4-inch wafer scale MoS_2 catalysts.

Supplementary Note 1: Experimental details

Device fabrication procedure

The MoS_2 electrochemical devices were fabricated using as-grown type-I and type- III monolayer MoS_2 on Si/SiO_2 substrates. PMMA was first spin-coated onto the Si/SiO_2 surface at 4000 rpm for 60 seconds. The samples were then prebaked at 180°C for 90 seconds. Then, e-beam lithography (EBL) was conducted to pattern gold contact, followed by development, metal deposition, and lift-off processes. Ti/Au (3nm/40nm) was deposited as metal electrodes using an e-beam evaporator at a deposition rate of 0.7 Å/s under high vacuum conditions (10^{-7} Torr). Note that type- III MoS_2 sample need to perform one more step that the films should be patterned to ribbons using EBL and oxygen plasma etching. Thus each ribbon can be tested separately for subsequent comparison (Fig S2). Finally, to eliminate the influence of electrolyte and to avoid electrochemical reactions on the metal electrodes, another layer of PMMA was then deposited on the MoS_2 device with spin coating. A smaller window that only exposes the basal plane was opened by EBL. The device measurements were performed using electrochemical workstation (Autolab PGSTAT 302N) in a self-developed probe station system (Fig S2), platinum wire and Ag/AgCl electrodes are used as counter and reference electrode, respectively.

Hydrogenation of heterophase MoS_2

To hydrogenate the heterophase MoS_2, the heterophase MoS_2 samples were directly immersed in hydrogen plasma at 0.3 Torr with 30W input powers. The hydrogen plasma was ignited a RF coil with the frequency of 13.56 MHz in our remote-plasma enhanced chemical vapour deposition (rPECVD) system. The duration of plasma treatment was set at 5 min with a continuous Hydrogen flow rate of 100 sccm and vacuum pumping. The treatment was carried out at room temperature.

Local electronic probe of hydrogen adsorption of heterophase MoS_2

Scanning tunneling microscopy/spectroscopy (STM/STS) was used to obtain the

hydrogenation of heterophase MoS_2. STM experiments were performed ex situ in a combined nc-AFM/STM system (Createc, Germany) at 77 K with base pressure <7×10-9 Pa. Electrochemically etched W-tips were cleaned by alternative annealing and sputtering before the experiments, and further by controlled field-emission and voltage-pulse procedures during the scanning. Bias voltage refers to the sample voltage with respect to the tip. All of the STM topographic images were obtained in constant-current mode. The scanning tunnelling spectroscopy (STS) dI/dV spectra were acquired using lock-in detection of the tunnelling current by adding a 5 mVrms modulation at 481 Hz to the sample bias.

Growth of graphene films on pristine monolayer MoS_2

Growth of nanographene was carried out in our homemade remote-plasma enhanced chemical vapour deposition (rPECVD) system.[3-5] Inductively coupled plasma is generated by a RF coil with the frequency of 13.56 MHz. The precursor is methane (flow rate: 30sccm) that can be dissolved into radicals in plasma for direct growth. Continuous graphene films were directly deposited on monolayer MoS_2 supported by SiO_2/Si substrate. The growth temperature is ~450℃ which may not affect the quality of most monolayer MoS_2. During deposition, the pressure in chamber was kept constant at ~0.2 Torr.

The grain size of as-grown graphene is much smaller than grown by CVD method, at nm scale. The AFM and SEM images of as-grown graphene on monolayer MoS_2 is shown in Fig. S6. As the growth time is 2h, the grain size of graphene varies from a few to tens of nanometers and the thickness of graphene/MoS_2 films is around 1.9 nm.

Fabrication process of heterophase-MoS_2/graphene/Au catalytic electrode

The heterophase-MoS_2/graphene (Gr)/Au electrode were fabricated based on a direct peel-off process (Fig. S5). Firstly, graphene was prepared on as-grown monolayer MoS_2 as mentioned above. The graphene is about 2 layers. Then 20nm-thick gold film was deposited on the surface by e-beam evaporation. After deposition, a thermal release tape was sticked to the surface smoothly and then peeled off. Thus, the Au/Gr/MoS_2

films were separated from SiO_2/Si substrate. This is resulted from the competition of binding energy (*f*) between interfaces, in the present case, $f_{Au\text{-}graphene}$ and $f_{graphene\text{-}MoS2}$ lager than $f_{MoS2\text{-}Substrate}$.[6] Therefore, we achieved the three layer structures with the MoS_2 on the top. Finally, we use Argon plasma treatment to obtain heterophase MoS_2 aforementioned.

Raman characterization

The Raman measurement was performed with the excitation laser line of 532 nm using a Horiba Jobin Yvon Lab RAM HR-Evolution Raman system in ambient air environment. The power of the excitation laser line was kept well below 5 mW to avoid damage of MoS_2. The Raman scattering was collected by an Olympus 100 × objective (N.A. =0.9).

The Raman spectra of as-grown MoS_2 on Silicon substrate, pristine-MoS_2/nanographene/Au and Au substrate were shown in Fig. S6. Two typical Raman peaks of MoS_2 (A1g at ≈ 404.6 cm^{-1} and E_{2g} at ≈384.6 cm^{-1}) were observed with a frequency difference of ≈20cm^{-1} for the as-grown MoS_2 on silicon substrate, suggesting the monolayer feature of 2H-MoS_2.[6] Intriguingly, a blue shift (≈2 cm^{-1}) of the E_{2g} peak for MoS_2/nanographene/Au (≈382.6 cm^{-1}) is observed compared with that of MoS_2/Si, and both A_{1g} and E_{2g} peaks broaden together with the decrease of intensity. This is due to perturbations to the perfect 2H-phase lattice, resulting in poorly defined vibrational mode energies,[7] suggesting that the peel-off process changes the vibrational energies of the lattice.

X-ray photoelectron spectroscopy (XPS) characterization

XPS characterization was carried out to confirm the as-grown MoS_2/Gr films on SiO_2 using Kratos Analytical Axis Ultra system. The peaks around 229.1 and 232.3 eV correspond to the $Mo4^{+3}d_{5/2}$ and $Mo^{4+}3d_{3/2}$ components in 2H-MoS_2, as shown in Fig. s13a. And Fig. S13b shows the binding energies of 161.9 and 163.1 eV corresponding to the S $2p_{3/2}$ and $2p_{1/2}$ of MoS_2, respectively.[8] The C 1s peak (284.7eV) confirms graphene C-C network (Fig. S13c).

Growth of wafer-scale monolayer 2H-MoS_2

The wafer-scale MoS_2 growth was performed in a three-temperature-zone chemical vapor deposition (CVD) chamber. S (Alfa Aesar, 99.9%, 4 g) and MoO_3 (Alfa Aesar, 99.999%, 50 mg) powders, loaded in two separate inner tubes, were used as sources and placed at zone-I and zone-II, respectively, and 4 in. sapphire wafers were loaded in zone-III as the substrates. During the growth, the two inner tubes were flowed with Ar (gas flow rate 100 sccm) and Ar/O_2 (gas flow rate 75/3 sccm) as carrying gases, respectively. During the growth, the temperatures for the S source, MoO_3 source, and wafer substrate are 115, 540, and 900 °C, respectively. For a typical growth, the growth duration is ∼40 min, and the pressure in the growth chamber is ∼1Torr.

Supplementary Note 2: Theoretical Approach

Computational details

First-principles calculations based on Density Functional Theory (DFT) were carried out by using the Vienna Ab initio Simulation Package (VASP).[9,10] The interactions between valence electrons and ions were treated with the projector-augmented wave (PAW) method.[11] The exchange-correlation interactions were described by generalized gradient approximation (GGA)[12] with the Perdew−Burke−Ernzerhof (PBE) functional.[13] The electron wave functions were expanded in a plane-wave basis set with cutoff energy of 520 eV. The convergence criterion for residual force on each atom during structure relaxation was set to 0.02 eV/Å. and the geometries were relaxed to minimize the total energy of the system until a precision of 10^{-4} eV was reached.

Hydrogen adsorption free energy

We study 4 kinds of grain boundaries of the MoS_2 monolayer , including 21.8°-tilt composed of 5-7rings , 60°-tilt composed 4-4 rings , 4-8rings and 6-8rings as shown in Fig. 3. The periodic structure model of grain boundaries is complicated to build, so we

used different sizes of supercell and k-point mesh to simulate grain boundaries. All structure are fully relaxed and the vacuum spaces in all supercells were larger than 12 Å above the MoS_2 plane to avoid any artificial interaction.

We used a supercell to simulate the 1T and 2H phase boundaries. As is well known, there are three types of boundaries between 1T and 2H phases of MoS_2 of which two of them are shown. These two boundaries are both along the zigzag boundaries and the third one is along the armchair edges. Based on the results of STM measurements, only zigzag boundaries were found in our samples. Thus, only zigzag boundaries were taken into account here. It can be seen that in one ideal boundary, the Mo atoms are seven-coordinated and in another ideal boundary, the Mo atoms are five-coordinated. In the supercell a vacuum space of >12 Å was used to avoid the interaction between periodical images. Only Γ-point was used for the Monkhorst −Pack k-point mesh. The S atoms in the bottom face are fixed to simulate the effect of the support and the HER catalytic property of the upper face is studied by the calculations of H adsorption energies with equation:

$$\Delta E_H=\frac{1}{n}[E(surf+nH)-E(surf)-\frac{n}{2}\bullet E(H_2)] \quad (1)$$

Where n is the number of the adsorbed H atoms in the supercell, E(surf+nH) and E(surf) are the energies of the supercells with and without adsorbed H. $E(H_2)$ is the energy of a free H_2 molecule. To more conveniently describe the HER activity of the catalyst, the Gibbsfree energy of the adsorbed H were further calculated following the method presented in Ref.14,i.e.,

$$\Delta G_{H^*} =\Delta E_H+\Delta E_{ZPE}-T\Delta S_H \quad (2)$$

Where ΔE_H is the H adsorption energy calculated by Eq.1 and ΔE_{ZPE} is the difference in zero point energy between the adsorbed H and the gas phase. The vibrational entropy in the adsorbed state is small and the entropy difference $\Delta S_H \cong -1/2S^0_{H2}$, where$S^0_{H2}$ is the entropy of H_2 in the gas phase at standard conditions as discussed in Ref.14, corresponding to 1 bar ofH_2 at 300 K. The contribution of the catalyst to ΔE_{ZPE} and ΔS_H are small and can be neglected. Our calculations give ΔE_{ZPE} to be 0.08 eV and $T\Delta S_H$ to be -0.21 eV at room temperature. So we have $\Delta G_{H^*}=\Delta E_H+$

0.29 eV. The calculated lattice constant of the hexagonal 2H phase is 3.19Å. The supercells employed in this study have rectangular geometry, with 4 x $4\sqrt{3}$ unit cells, which include 32 Mo and 64 S atoms respectively.

The ΔG_{H*} on the Pt(111)(-0.18eV) surface varies slightly on H coverage, as the reference for assessing the catalytic activities of different sites on MoS_2. As is well established, the calculated ΔE_H and ΔG_{H*} depend on the functionals chosen. Thus, we do not focus on the exact values of the calculated ΔG_{H*} but on the comparison between the value for the Pt (111) surface and them. To confirm the reference value, we calculated the ΔG_{H*} of Mo-edge (-0.115eV), differing about ~0.06eV with Pt (111) surface, which is in agreement with the result before. Moreover, the supercells for the calculations of ΔG_{H*} on pristine 2H-phase and pristine 1T-phase have the same sizes as for the calculations on phase boundaries. The grain boundaries with different structures also adsorbed one H on one S atom after structural optimization.

In our calculations, the size of the supercell is limited due to the limitations in computing power and crystalline geometry. We had to include two boundaries in one model and the distance between the two boundaries is limited to ~1nm. However, the trend revealed by the present model is general. The 2H-phase is too inert to adsorb H and the 1T-phase is too active to adsorb H atoms moderately as required by HER. At the phase boundaries, due to the atomic relaxations, the S atoms from both phases can become active sites. In this case, the active sites are on the basal plane and not limited to the edges of MoS_2 and the density of the active sites can be significantly increased.

Atomic structure with S-vacancies

Each unit cell consists of 16 Mo atoms and 32 S atoms on the surface. Assuming that S-vacancies are only formed on the surface. With the %S-vacancy being defined as (number of S-vacancies)/ (number of total S atoms).

Supplementary References

1 Zhang, J. *et al.* Scalable growth of high-quality polycrystalline MoS_2 monolayers

on SiO_2 with tunable grain sizes. *ACS nano* **8**, 6024-6030 (2014).

2 Zhu, J. *et al.* Argon Plasma Induced Phase Transition in Monolayer MoS_2. *Journal of the American Chemical Society* **139**, 10216-10219 (2017).

3 Zhang, L. *et al.* Catalyst-free growth of nanographene films on various substrates. *Nano Research* **4**, 315-321 (2011).

4 Yang, W. *et al.* Growth, characterization, and properties of nanographene. *Small* **8**, 1429-1435 (2012).

5 Meng, J., Shi, D. & Zhang, G. A review of nanographene: growth and applications. *Modern Physics Letters B* **28**, 1430009 (2014).

6 Zhao, J. *et al.* Patterned Peeling 2D MoS_2 off the Substrate. *ACS Applied Materials & Interfaces* **8**, 16546-16550 (2016).

7 Li, H. *et al.* Activating and optimizing MoS_2 basal planes for hydrogen evolution through the formation of strained sulphur vacancies. *Nature materials* **15**, 48-53 (2016).

8 Laursen, A. B., Kegnæs, S., Dahl, S. & Chorkendorff, I. Molybdenum sulfides—efficient and viable materials for electro-and photoelectrocatalytic hydrogen evolution. *Energy & Environmental Science* **5**, 5577-5591 (2012).

9 Kresse, G. & Furthmüller, J. Efficient iterative schemes for ab initio total-energy calculations using a plane-wave basis set. *Physical review B* **54**, 11169 (1996).

10 Kresse, G. & Furthmüller, J. Efficiency of ab-initio total energy calculations for metals and semiconductors using a plane-wave basis set. *Computational materials science* **6**, 15-50 (1996).

11 Blöchl, P. E. Projector augmented-wave method. *Physical review B* **50**, 17953 (1994).

12 Perdew, J. P., Burke, K. & Ernzerhof, M. Generalized gradient approximation made simple. *Physical review letters* **77**, 3865 (1996).

13 Perdew, J. P., Ernzerhof, M. & Burke, K. Rationale for mixing exact exchange with density functional approximations. *The Journal of chemical physics* **105**, 9982-9985 (1996).

14 Nørskov, J. K. *et al.* Trends in the exchange current for hydrogen evolution. *Journal of the Electrochemical Society* **152**, J23-J26 (2005).
