# Supplementary material for: Boundary activated hydrogen evolution reaction on monolayer MoS2
Source: Nat Commun. 2019 Mar 22;10:1348. doi: 10.1038/s41467-019-09269-9 (PMC6430794; doi:10.1038/s41467-019-09269-9)
Supplement: Supplementary file 2 — Description of Additional Supplementary Files [file 41467_2019_9269_MOESM2_ESM.pdf]

Description of Additional Supplementary Files

File Name: Supplementary Movie 1
Description: **Wafer-scale multi-hierarchy MoS_2 catalysts for HER.** The video of the catalytic HER activity in 0.5-M H_2SO_4 from a multi-hierarchy MoS_2 catalyst with a size of 4 inches in diameter.
